# Potential osteomyelitis biomarkers identified by plasma metabolome analysis in mice

**DOI:** 10.1038/s41598-020-57619-1

**Published:** 2020-01-21

**Authors:** Norihiro Isogai, Yuta Shiono, Tetsuya Kuramoto, Kenji Yoshioka, Hiroko Ishihama, Haruki Funao, Masaya Nakamura, Morio Matsumoto, Ken Ishii

**Affiliations:** 1 Department of Orthopaedic Surgery, Keio University School of Medicine, Shinjuku, Tokyo, Japan; 2 Department of Orthopaedic Surgery, International University of Health and Welfare (IUHW) Mita Hospital, Tokyo, Japan; 3 Department of Orthopaedic Surgery, School of Medicine, International University of Health and Welfare (IUHW), Chiba, Japan

**Keywords:** Metabolomics, Diagnostic markers

## Abstract

Osteomyelitis, which often arises from a surgical-site infection, is a serious problem in orthopaedic surgery. However, there are no specific biomarkers for osteomyelitis. Here, to identify specific plasma biomarkers for osteomyelitis, we conducted metabolome analyses using a mouse osteomyelitis model and bioluminescence imaging. We divided adult male pathogen-free BALB/C mice into control, sham-control, and infected groups. In the infected group, a bioluminescent *Staphylococcus aureus* strain was inoculated into the femur, and osteomyelitis was detected by bioluminescence imaging. We next analysed the metabolome, by comprehensively measuring all of the small molecules. This analysis identified 279 metabolites, 12 of which were significantly higher and 45 were significantly lower in the infected group than in the sham-control and control groups. Principal component analysis identified sphingosine as the highest loading factor. Several acyl carnitines and fatty acids, particularly ω-3 and ω-6 polyunsaturated fatty acids, were significantly lower in the infected group. Several metabolites in the tricarboxylic acid cycle were lower in the infected group than in the other groups. Thus, we identified two sphingolipids, sphinganine and sphingosine, as positive biomarkers for mouse osteomyelitis, and two components in the tricarboxylic acid cycle, two-oxoglutarate and succinic acid, as negative biomarkers.

## Introduction

Osteomyelitis, a serious problem in orthopaedic surgery, often arises from a surgical-site infection, which is mostly caused by *Staphylococcus aureus*^1^. Clinical signs and conventional laboratory markers, such as increases in the white blood cell count, erythrocyte sedimentation rate, C-reactive protein, and inflammatory cytokines (e.g. interleukin 1β, 6) are widely used to detect postoperative infection, but these markers are also elevated by non-infectious inflammation. Although several candidates have been investigated^2^, no specific biomarkers for diagnosing osteomyelitis have been identified^3^.

Metabolome analysis, which involves the comprehensive measurement of all low-molecular-weight compounds present in a biological system, has been used to identify novel biomarkers for several diseases^4,5^. Capillary electrophoresis time-of-flight mass spectrometry (CE-TOFMS) and liquid chromatography time-of-flight mass spectrometry (LC-TOFMS) are powerful tools for determining a broad range of metabolites with different characteristics, and can examine thousands of metabolites^6,7^.

Previous reports showed several animal models of osteomyelitis^8–10^. Optical bioluminescence imaging (BLI) permits the real-time and noninvasive monitoring of cell growth and gene expression *in vivo*. BLI enables the sequential monitoring of visualized and quantified bacterial growth in a mouse osteomyelitis model^10^. The efficacy and reproducibility of this monitoring method has been reported for several mouse infection models^10–12^.

Although there are many potentially bacterial species that cause osteomyelitis, *Staphylococcus aureus* is by far the most commonly isolated microorganism in most types of osteomyelitis^1^. Therefore, a better definition of the mechanisms used by *Staphylococcus aureus* microorganisms to establish infection in the bone in animal models are helpful to facilitate early diagnosis and develop more effective therapeutic modalities for osteomyelitis^13^.

In this study, we aimed to identify specific plasma biomarkers of osteomyelitis caused by *Staphylococcus aureus*, using BLI and metabolome analyses, including both CE-TOFMS and LC-TOFMS, with a reproducible osteomyelitis mouse model.

## Methods

### Bioluminescent bacteria and inoculation

A bioluminescent bacterial strain of *Staphylococcus aureus*, Xen-29, was obtained from Caliper LS Co. (Hopkinton, MA) and cultured in Luria Bertani medium (Sigma-Aldrich Co., St Louis, MO) at 37 °C under ambient aeration with gentle agitation. The bacteria were selectively grown in medium containing 200 μg/ml kanamycin. *Staphylococcus aureus* Xen-29, derived from the parental strain American Type Culture Collection 12600, has a stable copy of a modified *Photorhabdus lumenscens lux* ABCDE operon, which encodes the enzymes responsible for the luminescent reaction. The bacterial bioluminescence does not require any added substrate to generate light, and constitutively emits a bioluminescent signal as long as the organism is viable. The bacterial samples were frozen and stored at −80 °C in Luria Bertani medium. The samples were thawed at 4 °C for 1 h prior to each experiment. Typically, bacterial viability was maintained at 4 °C for approximately 5 h after thawing^10,11^.

### Mouse osteomyelitis model

Thirty-six pathogen-free BALB/C adult male mice (12 weeks old; body weight 20 to 25 g) purchased from Sankyo Service (Shizuoka, Japan) were used in this study. The mice were assigned to three groups (infected, sham-control, and control groups; n = 12 each) and were maintained in our animal facility under specific-pathogen-free conditions^10^. The number of samples was determined by previous reports using metabolome analysis^14^.

In the infected group, mice were anesthetized with an intraperitoneal injection of butorphanol (5.0 mg/kg of body weight), medetomidine (0.4 mg/kg), and midazolam (2.0 mg/kg), and the skin on the left knee was shaved and sterilized with povidone iodine. A skin incision was made over the left knee, and the distal end of the femur was exposed through a lateral parapet arthrotomy with medial displacement of the quadriceps-patella complex. The distal end of the femur was perforated using a high-speed drill with a 0.5-mm sharp steel burr (Fine Science Tools Inc., Foster City, CA). Bioluminescent *Staphylococcus aureus* (1.0 × 10^8^ CFU in 1 μl of Luria Bertani medium) was inoculated into the medullary cavity of the femur using a Hamilton syringe. The burr hole was closed with bone wax, the dislocated patella was reduced, and the muscle and skin openings were closed by suture^10^. The animals were placed on a heating pad and monitored until they recovered. In the sham-control group, the mice underwent the same procedure but without bacterial inoculation. This study was performed in strict accordance with recommendations in the Guide for the Care and Use of Laboratory Animals of the National Institutes of Health. The protocol was approved by the Keio University Committee on the Ethics of Animal Experiments (Permit number 09108), and all experiments were approved by the Animal Care and Use Committee of Keio University.

### BLI

To monitor bacterial growth in the femur, we measured bacterial photon intensity (PI) by BLI immediately after surgery and on day 3 after the surgery. The mice were anesthetized via inhaled aerosolized isoflurane mixed with oxygen, placed on their back, and imaged for 5 min. To quantify bacterial luminescence, we defined and examined regions of interest (ROIs) in the inoculated area^10^. For BLI, we used a Caliper LS-Ivis Lumina cooled CCD optical macroscopic imaging system (Summit Pharmaceuticals International Co., Tokyo, Japan)^15^ to detect inoculated bacteria that emit a bacterial bioluminescent signal through the tissues of a living animal. Photon emissions of the bacterial bioluminescent signal were captured, converted to false-colour photon-count images, and quantified with Living Image software version 3.0 (Caliper LS Co., Hopkinton, MA). Bacterial PI was expressed as photon flux in units of photons/s/cm^2^/sr.

### Serology

Blood samples were collected from an abdominal artery of mice under ether anaesthesia on day 3 after the operation. Samples were collected after the mice had fasted for 12 h. All samples were collected by one physician with experience in drawing this type of sample. Plasma was obtained by centrifuging the samples at 1200 rpm at 4 °C for 10 min. In each group, the two smallest plasma samples were excluded, the remaining samples were pooled, and 2 samples were combined to 1 specimen to make 5 specimens per group. The specimens were stored at −80 °C until use^16–20^. This study was performed in strict accordance with recommendations in the Guide for the Care and Use of Laboratory Animals of the National Institutes of Health.

### Metabolome analysis

To inactivate native enzymes, 50 μl of specimen was added to 450 μl of methanol at 0 °C. This extract solution was added to 500 μl of chloroform and 200 μl of Milli-Q water and then centrifuged at 2300 rpm at 4 °C for 5 min. The upper aqueous layer (400 μl) was centrifuged through a Millipore 5-kDa cut-off filter to remove proteins, and then centrifuged at 9100 rpm at 4 °C for 120 minutes. The filtrate was lyophilized, suspended in 25 μl of Milli-Q water, and analysed by CE-TOFMS.

In addition, 1500 μl of 1% formic acid acetonitrile was added to 500 μl of specimen, and the sample was centrifuged at 2300 rpm at 4 °C for 5 min. After solid-phase extraction to remove phospholipids, the filtrate was recovered and lyophilized, suspended in 100 μl of 50% isopropanol, and analysed by LC-TOFMS. Both CE-TOFMS and LC-TOFMS were performed in a facility at Human Metabolome Technologies (Tsuruoka, Japan)^5^.

### Statistical analysis

The peaks detected by CE-TOFMS and LC-TOFMS were processed with Master Hands ver.2.13.0.8.h (Keio University) to obtain m/z values, peak areas, and migration time (CE-TOFMS) or retention time (LC-TOFMS)^21^. The metabolic pathway map was provided using the public-domain software VANTED (Visualization and Analysis of Networks containing Experimental Data, Germany)^22,23^. Principal component analysis (PCA) and hierarchical clustering analysis (HCA) were performed using SampleStat ver.3.14 and PeakStat ver.3.18 (Human Metabolome Technologies). PCA is the most widely used dimension-reducing technique for analysing the large datasets involved in metabolome analysis^24^. A heatmap was generated for the HCA, with red and green indicating high and low concentrations, respectively. All values were presented as the mean ± standard deviation. We considered a p value less than 0.05 to be significant (Welch’s *t* test).

## Results

### Bacterial PI

**Figure 1:**
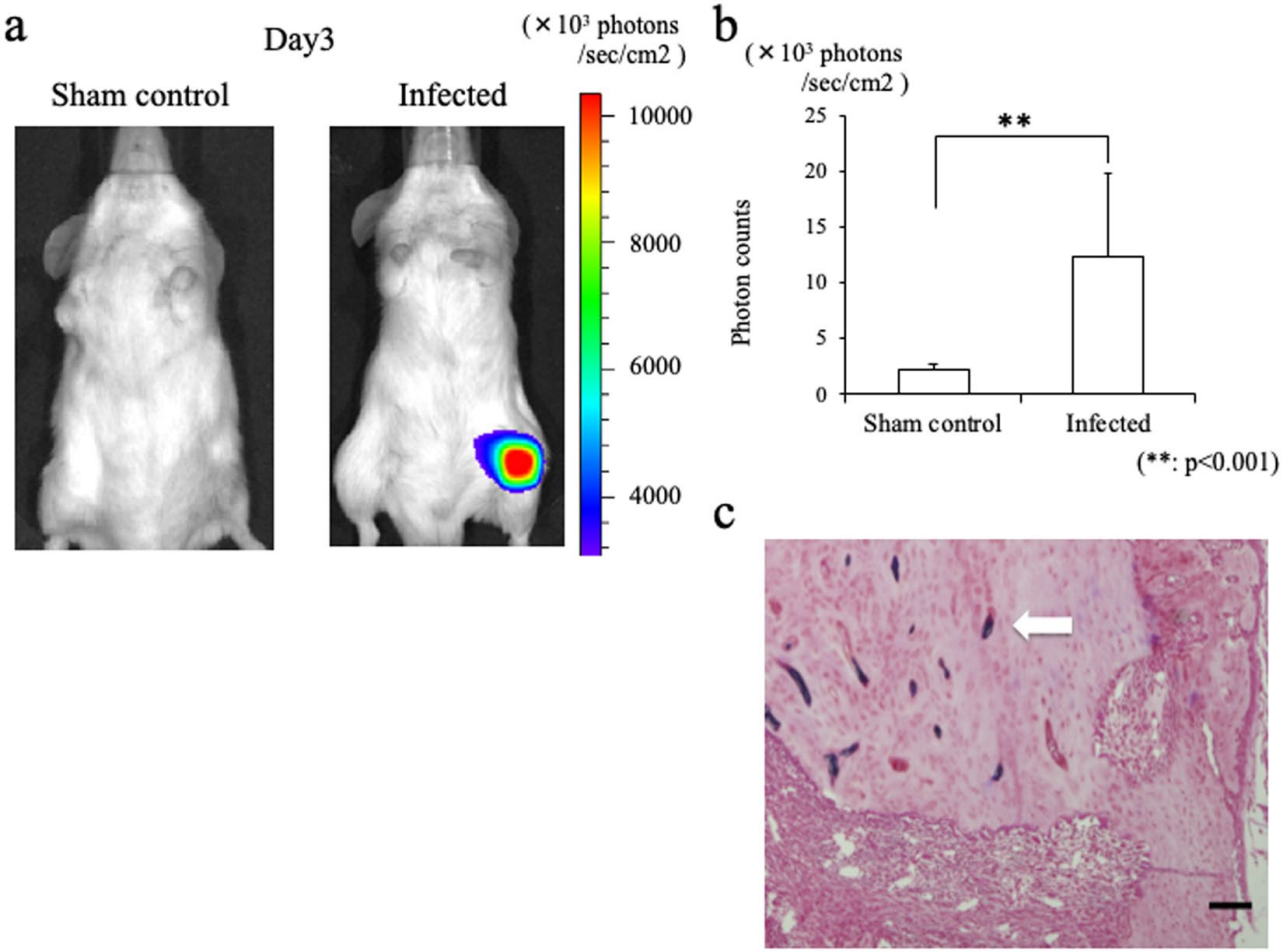
Osteomyelitis model mice infected with a bioluminescent *Staphylococcus aureus* strain. (**a**) Representative image showing a stable luminescent signal at the left femur of an infected mouse on day 3 after surgery. (**b**) Bar graph showing photon counts for the front-view ROI. The mean bacterial PI was significantly higher in the infected group than in the sham-control group (**p < 0.001). (**c**) The histology of femur from infected mice day 3 after surgery. Gram-stain-positive bacteria are observed in the bone-marrow space. Bars = 100 μm.

On day 3 after surgery, stable luminescent signals were detected in all surviving mice in the infected group (Fig. 1a). The mean bacterial PI in the infected group was 12.3 ± 7.4 × 10^3^ photons/s/cm^2^/sr on the front view and 12.2 ± 5.1 × 10^3^ on the lateral view. In contrast, the mean bacterial PI in the sham-control group was 2.3 ± 0.4 × 10^3^ photons/s/cm^2^/sr on the front view and 2.7 ± 0.5 × 10^3^ on the lateral view. The highest PI in the sham-control group was less than 3.5 × 10^3^ photons/s/cm^2^/sr on both views. The bacterial PI was significantly higher in the infected group (p < 0.001; Fig. 1b). In addition, the presence of *Staphylococcus aureus* was confirmed in the histology of femur from the mice in the infected group day 3 after surgery (Fig. 1c).

### Metabolome analysis

**Figure 2:**
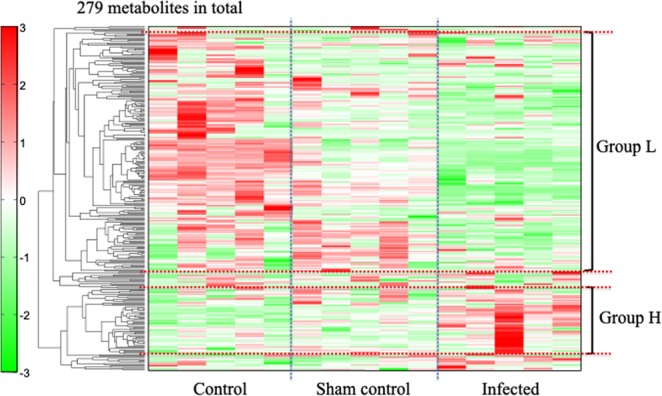
Hierarchical clustering analysis of the metabolites in the Control, Sham-control, and Infected groups. In Group H, 66 metabolites (red bars) were elevated in the infected group compared to the sham or control groups, and 12 of them were significantly higher in the infected group (p < 0.05). Of 195 Group L metabolites (green bars), which decreased in the infected compared to the sham-control or control groups, 45 were significantly lower in the infected group (p < 0.05).

We detected 279 metabolites that differed between the infected group and the sham-control and control group (191 by CE-TOFMS and 88 by LC-TOFMS) among the 1200 molecules we measured. An HCA heat map showed 66 metabolites that increased in the infected group (Fig. 2, Group H), 12 of which were significantly higher in the infected group than in either the sham-control or control group (p < 0.05). We found 195 metabolites that decreased in the infected group (Fig. 2, Group L), 45 of which were significantly lower in the infected group than in either the sham-control or control group (p < 0.05). Metabolites that increased or decreased significantly in the infected group were classified according to the Human Metabolome Database (HMDB; www.hmdb.ca), as shown in Table 1.

**Table 1:** Classification of 12 Group H and 45 Group L metabolites according to the Human Metabolome Database (HMDB; www.hmdb.ca).

Pathway Label	HMDB:class(subclass)
	Pathway Index^§^
**Group H: 12 metabolites**	
1-Methylhistamine	Amines
*N*-Glycolylneuraminic acid	Carbohydrates and carbohydrate conjugates
Gln	Carboxylic acids and derivatives
2-Aminobutyric acid	Carboxylic acids and derivatives
Homocitrulline	Carboxylic acids and derivatives
Glu	Carboxylic acids and derivatives
Guanidoacetic acid	Carboxylic acids and derivatives
Asp	Carboxylic acids and derivatives
N8-Acetylspermidine	Carboxylic acids and derivatives
γ-Butyrobetaine	Fatty Acyls
Sphingosine	Sphingolipids
Sphinganine	Sphingolipids
**Group L: 45 metabolites**	
Oleoyl ethanolamine	Amines
Sarcosine	Carboxylic acids and derivatives
Ornithine	Carboxylic acids and derivatives
N-Acetylleucine	Carboxylic acids and derivatives
Betaine	Carboxylic acids and derivatives
4-Acetamidobutanoic acid	Carboxylic acids and derivatives
Gly	Carboxylic acids and derivatives
Citrulline	Carboxylic acids and derivatives
Citric acid	Carboxylic acids and derivatives
cis-Aconitic acid	Carboxylic acids and derivatives
Isocitric acid	Carboxylic acids and derivatives
Thiamine	Diazines
Palmitoylcarnitine	Fatty Acyls
Isobutyrylcarnitine	Fatty Acyls
AC(17:1)	Fatty Acyls
AC(16:1)	Fatty Acyls
AC(14:1)	Fatty Acyls
AC(12:0)	Fatty Acyls
Palmitoleic acid	Fatty Acyls
Palmitic acid	Fatty Acyls
Myristic acid	Fatty Acyls
Malic acid	Fatty Acyls
Indole-3-carboxaldehyde	Fatty Acyls
FA(22:5)	Fatty Acyls
FA(22:4)	Fatty Acyls
FA(20:3)	Fatty Acyls
FA(19:0)	Fatty Acyls
FA(17:1)	Fatty Acyls
FA(17:0)	Fatty Acyls
FA(14:2)	Fatty Acyls
FA(14:1)-2	Fatty Acyls
FA(14:1)-1	Fatty Acyls
Ethyl arachidonate	Fatty Acyls
cis-8,11,14-Eicosatrienoic acid	Fatty Acyls
Arachidonic acid	Fatty Acyls
3-Hydroxytetradecanoic acid	Fatty Acyls
3-Indoxylsulfuric acid	indoles and derivatives
Linolenic acid	Lineolic acids and derivatives
Linoleic acid	Lineolic acids and derivatives
7,8-Dihydrobiopterin	Pteridines and derivatives
Uridine	Pyrimidine nucleosides
Hypotaurine	Quaternary ammonium salts
Taurodeoxycholic acid	Steroids and steroid derivatives
21-Deoxycortisol	Steroids and steroid derivatives
N-Ethylmaleimide_ + H2O	Vinyl halides

**Figure 3:**
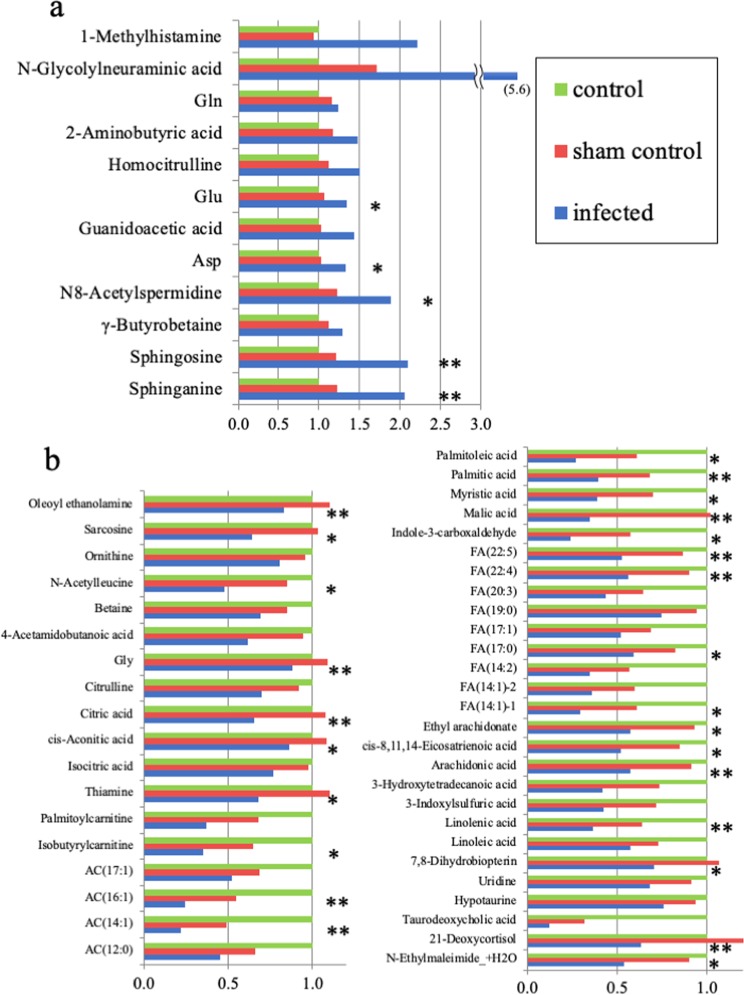
Plasma concentration of metabolites in the H and L Groups. Data are expressed as concentration relative to control for the sham control and the infected group. (**a**) Plasma concentrations of 12 Group H metabolites that were significantly higher in the infected group than the sham-control group (p < 0.05); the p values were lowest for sphingosine and sphinganine (p < 0.001). Carboxylic acids or derivatives with 7 metabolites were the most pathway label in the Group H. (**b**) Plasma concentrations of 45 Group L metabolites that were significantly lower in the infected group than in the sham-control group (p < 0.05); the p values were lowest for glycine and oleoyl ethanolamine (p < 0.001). Fatty acyls with 24 metabolites were the most pathway label in the Group L. The P values for each row are: no mark, p < 0.05; *p < 0.01, and **p < 0.001.

Group H consisted of 1 amine, 1 carbohydrate or carbohydrate conjugate, 7 carboxylic acids or derivatives, 1 fatty acyl, and 2 sphingolipids (Table 1). In comparisons between the infected and sham-control groups, the lowest p values were for the 2 sphingolipids, sphingosine (p < 0.001) and sphinganine (p < 0.001) (Fig. 3a). The individual plasma concentrations of sphingosine and sphinganine revealed that there were significantly higher in the infected group, compared with both the control group and the sham control group (p < 0.01 each: Welch’s *t* test) (Table 2).

**Table 2:** Individual plasma concentration of sphingosine and sphinganine relative to control average.

Sphingoshine	Control	Sham	Infected
1	1.01	1.20	1.95
2	0.92	1.18	2.11
3	0.87	1.06	2.26
4	1.06	1.25	1.75
5	1.06	1.36	2.38
Average	1.00	1.21	2.09
**Sphinganine**			
1	1.01	1.31	1.93
2	0.97	1.08	2.22
3	0.81	1.07	2.22
4	1.04	1.18	1.70
5	1.18	1.47	2.23
Average	1.00	1.22	2.06

There were significantly higher in the infected group, compared with both the control group and the sham control group (p < 0.01 each: Welch’s *t* test).

Group L consisted of 1 amine, 10 carboxylic acids or derivatives, 1 diazine, 24 fatty acyls, 1 indole or derivative, 2 lineolic acids or derivatives, 1 pteridine or derivative, 1 pyrimidine nucleoside, 1 quaternary ammonium salt, 2 steroids or steroid derivatives, and 1 vinyl halide (Table 1). Of the lineolic acids and derivatives, 2 were significantly lower in the infected group: linoleic acid (p < 0.05) and linolenic acid (p < 0.001). The fatty acyls included 20 types of acylcarnitine (AC), of which 6 were significantly lower in the infected group, as follows: palmitoylcarnitine, (p < 0.01) isobutylcarnitine (p < 0.05), AC(17:1) (p < 0.05), AC(16:1) (p < 0.001), AC(14:1) (p < 0.001), and AC(12:0) (p < 0.05). The fatty acyls also included 38 types of fatty acids (FAs), of which 18 were significantly lower in the infected group, as follows: palmitoleic acid (p < 0.01), palmitic acid (p < 0.001), myristic acid (p < 0.01), malic acid (p < 0.001), indole-3-carboxaldehyde (p < 0.01), FA(22:5) (p < 0.001), FA(22:4) (p < 0.001), FA(20:3) (p < 0.05), FA(19:0) (p < 0.05), FA(17:1) (p < 0.05), FA(17:0) (p < 0.01), FA(14:2) (p < 0.05), FA(14:1)-2 (p < 0.05), FA(14:1)-1 (p < 0.01), ethyl arachidonate (p < 0.01), cis-8,11,14-eicosatrienoic acid (p < 0.01), arachidonic acid (p < 0.001), and 3-hydroxytetradecanoic acid (p < 0.05). Of the carboxylic acids and derivatives, the following were significantly lower in the infected group (Fig. 3b): sarcosine (p < 0.01), ornithine (p < 0.05), N-acetylleucine (p < 0.01), betaine (p < 0.05), 4-acetamidobutanoic acid (p < 0.05), glycine (p < 0.001), citrulline (p < 0.05), citric acid (p < 0.001), cis-aconitic acid (p < 0.01), and isocitric acid (p < 0.05).

Some metabolites of the tricarboxylic acid (TCA) cycle were depleted below the limit of detection in the infected group: two-oxoglutarate (2-OG), succinic acid, nicotinamide adenine dinucleotide (NAD^+^), and fumaric acid. In contrast, 2-OG was detected in all 5 sham-control and all 5 control specimens, succinic acid in all sham-control and 3 control specimens, and NAD^+^ in one sham-control and 3 control specimens. Fumaric acid was detected in one control specimen.

### PCA

**Figure 4:**
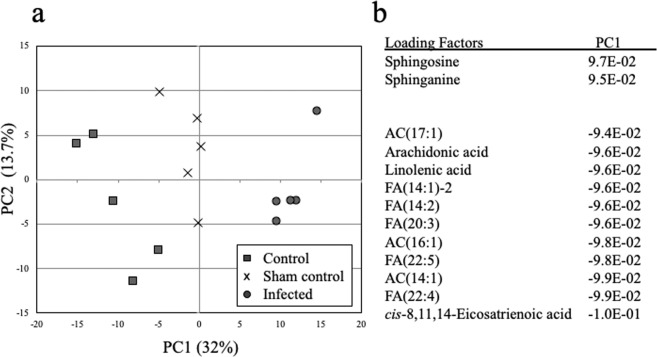
Principal component analysis of metabolites to identify the most important metabolites with the loading factor. (**a**) Principal component analysis showed that the three treatment groups were tightly clustered along the PC1 axis. (**b**) Loading factors of metabolites. Sphingosine was the highest and cis-8,11,14-eicosatrienoic acid was the lowest loading factor of the PC1 axis.

We used PCA to examine the metabolic effects of osteomyelitis. A PCA score plot showed that each of the three groups was tightly clustered along the PC1 axis (Fig. 4a). The highest loading factor for the PC1 axis in the H group was sphingosine (0.097), and the lowest loading factor of the PC1 axis in the L group was cis-8,11,14-eicosatrienoic acid (−0.1; Fig. 4b).

## Discussion

We used a mouse infection model that is reproducible and suitable for evaluating the pathophysiology of osteomyelitis, since the bacterial bioluminescence can be visualized and quantified immediately prior to sacrifice^8–10^. Because the blood samples of the infected and control mice are compositionally homogeneous, the plasma metabolome analysis provides more accurate results than those from other infection models. PCA revealed that the three treatment groups were tightly clustered along the PC1 axis, indicating that the results accurately reflected the effect of osteomyelitis with high reproducibility.

Sphingolipids are bioactive lipids that are involved in cellular signalling and regulatory functions^25^. Sphingolipids have been implicated as potent mediators in several inflammation and diseases, including cancers, inflammatory diseases and injury^25–30^. In addition, antimicrobial activity of sphingosine against *Staphylococcus aureus* was reported^27^, and lack of sphingosine induced pulmonary *Staphylococcus aureus* infection^28^. Therefore, sphingosine had an important role for *Staphylococcus aureus* infection as both a mediator and a resistant. Although sphingosine is reported to exert antimicrobial activity in infected areas of the skin and lung^27–30^, to the best of our knowledge, this is the first report describing a correlation between sphingosine and osteomyelitis. Moreover, the sphingomyelin cycle is an important sphingolipid pathway, and its turnover is so rapid that ceramide mass levels return to baseline within just 4 h^31^. In the present study, the sphingosine and sphinganine levels were significantly higher in the infected group than in the sham-control group 3 days after surgery (p < 0.001). Our results indicate that both infection and surgical stress can activate the sphingomyelin cycle; however, the rapid turnover of the sphingomyelin cycle can help to distinguish limited time stress such as operative stress from persistent stress such as osteomyelitis. Therefore, sphingosine and sphinganine are candidate positive biomarkers for osteomyelitis especially caused by *Staphylococcus aureus* in the early phase.

Omega-3 polyunsaturated fatty acids (ω-3 PUFAs), such as α-linolenic acid, eicosapentaenoic acid, and docosahexaenoic acid, have well-documented anti-inflammatory properties^32–38^, and potential benefits of supplementing the diet with ω-3 PUFAs have been reported^35,36,39^. In the initial inflammatory response, the mobilization of eicosapentaenoic acid and docosahexaenoic acid from the circulation to inflammation sites requires the conversion of these acids to resolvins, which control excessive neutrophil infiltration, protect organs, and promote the resolution of the inflammation^34^. Therefore, the plasma ω-3 PUFA levels are inversely correlated with infection^35^. In contrast, ω-6 PUFAs, such as linoleic and arachidonic acids, are precursors of eicosanoids (prostaglandins and leukotrienes)^40,41^. In our study, although linolenic acid (an ω-3 PUFA) and linoleic acid (an ω-6 PUFA) were in the L group, no specific changes were observed in other ω-3 or ω-6 PUFAs. Since PUFAs are nutritionally essential FAs, the ω-3 and ω-6 PUFAs are correlated with dietary intake like other FAs^42,43^. It would therefore be inappropriate to measure ω-3 and ω-6 PUFAs as specific osteomyelitis biomarkers.

In AC pathway, carnitine palmitoyltransferase-II releases fatty acyl coenzyme A (CoA) and free carnitine in mitochondria^44,45^. Several disorders associated with immune responses such as autoimmune diseases, chronic fatigue syndrome or infection reduce the pool of carnitines in the patient’s tissues or serum^46^. In other words, these situations accelerate β-oxidation, defined as the oxidation of fatty acyls to acetyl CoA. Elevated β-oxidation generates more adenosine triphosphate but decreases fatty acyl levels in the circulation^47^. Our results revealed that osteomyelitis also reduces the serum AC and FA levels by accelerating β-oxidation.

**Figure 5:**
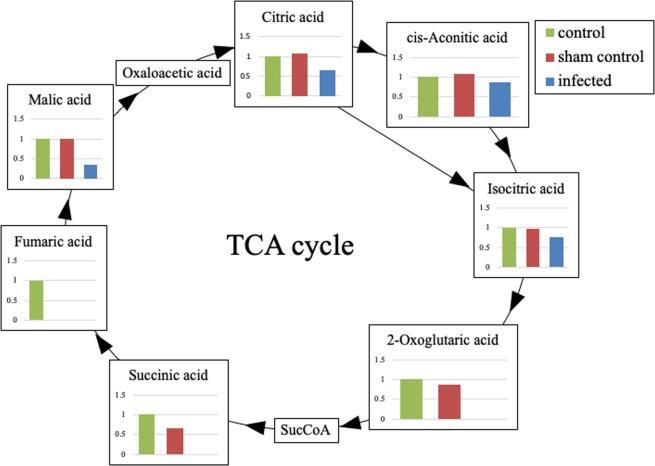
The TCA cycle. All metabolites in the TCA cycle were decreased or depleted in the infected group. In particular, four metabolites downstream of 2-OG (2-OG, succinyl CoA, succinic acid, and fumaric acid) fell below measurable limits in the infected group. The metabolic pathway from isocitric acid to 2-OG was strongly inhibited in the infected group according to the depletion of NAD^+^ from accelerated β-oxidation. The value of each metabolites is the concentration relative to the control.

Interestingly, in the infected group, all metabolites in the TCA cycle were decreased or depleted (Fig. 5). These molecules included malic acid, which is a fatty acyl, and citric and isocitric acid, which are carboxylic acids or derivatives. Malic acid, citric acid, and isocitric acid, that are upstream of isocitric acid, were significantly lower in the infected group (p < 0.05). Four metabolites downstream of 2-OG—2-OG, succinyl CoA, succinic acid, and fumaric acid—fell below measurable limits in the infected group. To the best of our knowledge, this is the first report that the metabolites in the TCA cycle were significantly decreased by osteomyelitis. Our results showed that the transfer from isocitric acid to 2-OG was inhibited in the infected group. This pathway is the oxidation reaction of NAD^+^ in the presence of NAD^+^-specific isocitrate dehydrogenase, which is inhibited by NADH^48,49^. In the present study, NAD^+^ fell below measurable limits only in the infected group. This depletion of NAD^+^ appeared to result from accelerated β-oxidation, since β-oxidation reduces NAD^+^ in a manner dependent on palmitoyl CoA^50^. Osteomyelitis may have accelerated β-oxidation, in turn decreasing all of the metabolites involved in the TCA cycle. Therefore, the metabolites in the TCA cycle, especially 2-OG and succinic acid, are potential negative biomarkers for osteomyelitis.

Thiamine in diazine is an essential coenzyme associated with the pyruvate decarboxylation that converts pyruvate into acetyl-CoA^51^. The pyruvate decarboxylation is accelerated by starvation like β-oxidation^52^. In the present study, thiamine was in the L group. Therefore, osteomyelitis may have also accelerated the pyruvate decarboxylation, decreasing thiamine like NAD^+^.

Histamine was reported to increase in peripheral blood concentration during parasite infection and virus infection^53,54^. In the present study, 1-Methylhistamine was in the H group. To the best of our knowledge, there are no reports about relationship between osteomyelitis and histamine. Therefore, bacterial infection might influence blood concentration of 1-Methylhistamine with any kind of inflammatory processes, although the mechanism is still not fully explained.

Previous reports showed other biomarkers for osteomyelitis such as inflammatory cytokines^55,56^ and antibody for *Staphylococcus aureus*^57–59^. However, to the best of our knowledge, there are no biomarkers that facilitate the diagnosis of osteomyelitis in the acute phase. For inflammatory cytokines, we previously evaluated the serum levels of interleukin-6 and interleukin-1β in this mouse model^10^. The mean serum levels of these biomarkers increased in the infected mice to the same extent in the sham control mice on day 3 after surgery, and they were significantly higher in the infected mice on day 7 after surgery^10^. Thus, the inflammatory cytokines could not distinguish between surgical-site infection and surgical stress at the acute phase after surgery. For this reason, several biomarkers detected in the present study are significantly useful, especially for early diagnosis of osteomyelitis.

There are several limitations to this study. First, we only analysed the *Staphylococcus aureus* infection model, but not other pathogens. Second, other inflammatory diseases such as rheumatoid arthritis were not evaluated. Although these new biomarkers are useful to diagnose *Staphylococcus aureus* infection, further study is needed to prove the specificity of these biomarkers for any other infectious diseases.

Taken together, of 1200 molecules measured in a mouse myelitis model, we identified 12 metabolites as candidate positive biomarkers for osteomyelitis, including the sphingolipids sphingosine and sphinganine. We also identified two candidate negative biomarkers for osteomyelitis, the TCA-cycle metabolites 2-OG and succinic acid. These new plasma biomarkers for osteomyelitis should improve the prognosis and treatment consistency for patients with postoperative osteomyelitis.

## Data Availability

The datasets analysed during the current study are available in the Metabolights repository, https://www.ebi.ac.uk/metabolights/MTBLS1398.
